# In-hospital antibiotic consumption in Lombardy, Italy (2017–2024): a retrospective analysis of regional administrative data

**DOI:** 10.1093/jac/dkag314

**Published:** 2026-09-07

**Authors:** Matteo Augello, Massimo Cartabia, Marina Azab, Camilla Tincati, Danilo Cereda, Olivia Leoni, Ida Fortino, Davide Mangioni, Giulia Renisi, Massimo Puoti, Pierluigi Plebani, Andrea Gori, Mauro Tettamanti, Alessandro Nobili, Alessandra Bandera, Giulia Marchetti

**Affiliations:** Clinic of Infectious Diseases and Tropical Medicine, San Paolo Hospital, ASST Santi Paolo e Carlo, Milan, Italy; Department of Health Sciences, University of Milan, Milan, Italy; Department of Health Policy, Mario Negri Institute for Pharmacologic Research IRCCS, Milan, Italy; Department of Health Policy, Mario Negri Institute for Pharmacologic Research IRCCS, Milan, Italy; Clinic of Infectious Diseases and Tropical Medicine, San Paolo Hospital, ASST Santi Paolo e Carlo, Milan, Italy; Department of Health Sciences, University of Milan, Milan, Italy; General Directorate for Health, Lombardy Region, Milan, Italy; General Directorate for Health, Lombardy Region, Milan, Italy; General Directorate for Health, Lombardy Region, Milan, Italy; Infectious Diseases Unit, Fondazione IRCCS Ca’ Granda Ospedale Maggiore Policlinico, Milan, Italy; Infectious Diseases Unit, Fondazione IRCCS Ca’ Granda Ospedale Maggiore Policlinico, Milan, Italy; Infectious Diseases Unit, ASST Grande Ospedale Metropolitano Niguarda, Milan, Italy; Department of Medicine and Surgery, University of Milano-Bicocca, Milan, Italy; Department of Electronics, Information and Bioengineering, Politecnico di Milano, Milan, Italy; Centre for Multidisciplinary Action for Global Health (MAGH), Milan, Italy; Infectious Diseases Unit II, Luigi Sacco Hospital, ASST Fatebenefratelli Sacco, Milan, Italy; Department of Biomedical and Clinical Sciences, University of Milan, Milan, Italy; Department of Health Policy, Mario Negri Institute for Pharmacologic Research IRCCS, Milan, Italy; Department of Health Policy, Mario Negri Institute for Pharmacologic Research IRCCS, Milan, Italy; Infectious Diseases Unit, Fondazione IRCCS Ca’ Granda Ospedale Maggiore Policlinico, Milan, Italy; Centre for Multidisciplinary Action for Global Health (MAGH), Milan, Italy; Department of Pathophysiology and Transplantation, University of Milan, Milan, Italy; Clinic of Infectious Diseases and Tropical Medicine, San Paolo Hospital, ASST Santi Paolo e Carlo, Milan, Italy; Department of Health Sciences, University of Milan, Milan, Italy

## Abstract

**Introduction:**

Antimicrobial stewardship (AMS) is essential to tackle antimicrobial resistance. Antibiotic consumption (AC) surveillance provides evidence to guide AMS interventions. This study investigated in-hospital AC trends in Lombardy, Italy (2017–2024), with particular focus on the impact of COVID-19.

**Methods:**

AC was expressed as DDDs per 100 bed days (absolute AC), relative proportions of Access/Watch/Reserve antibiotics (relative AC), Access-to-Watch index, and Access-to-(Watch + Reserve) index. Analyses were stratified by ward type—medical (MUs), surgical (SUs), and intensive care units (ICUs)—and timeframes: pre–COVID-19 (01/2017–02/2020), COVID-19 onset (02–03/2020), COVID-19 (03/2020–05/2023), and post–COVID-19 (05/2023–12/2024).

**Results:**

Despite a surge in total AC in 2017–2024, relative Access AC rose while relative Watch AC declined, resulting in an increase of the Access-to-Watch and Access-to-(Watch + Reserve) indexes, except in ICUs. A transient reversal of such favourable trends was observed at COVID-19 onset. Nevertheless, the WHO 2023 target of ≥60% total AC being Access was not achieved; furthermore, Access-to-Watch and Access-to-(Watch + Reserve) indexes constantly remained <1, except for SUs. A downward trend in Access-to-Watch and Access-to-(Watch + Reserve) indexes was registered in the post-pandemic period. Reserve AC increased across time, periods and wards, exceeding the expected levels obtained using projections from pre–COVID-19 data.

**Conclusions:**

These findings highlight substantial shifts in in-hospital AC in Lombardy during the period 2017–2024, with concerning increases in Reserve antibiotics and an enduring failure to meet the 2023 WHO target of ≥60% Access antibiotics especially in MUs and ICUs, underscoring the need for reinforced AMS policies in the post-pandemic era.

## Introduction

Antimicrobial resistance (AMR) is one of the most pressing global health challenges of the 21st century. In 2021, AMR was estimated to be associated with 4.71 million deaths globally, of which 1.14 million directly attributable to antimicrobial-resistant infections.^1^ In the EU, more than 35 000 deaths annually are estimated to result directly from antimicrobial-resistant infections, with a growing incidence of bloodstream infections (BSIs) caused by resistant bacteria reported in recent years,^2^ highlighting the urgent need for intensified public health actions against AMR.^3^

The prudent use of antimicrobials represents a cornerstone of AMR containment strategies. In 2023, the Council of the EU issued a recommendation calling on member states to strengthen actions to reduce antibiotic consumption (AC),^4^ including the achievement, by 2030, of at least 65% of antibiotic use from the WHO AWaRe Access group, which includes narrow-spectrum agents associated with a lower risk of promoting AMR.^5^ This objective is more ambitious than the WHO global 2023 target, which recommended a minimum of 60% Access AC,^6^ thereby reflecting an increasingly strengthened commitment to curbing AC.

In this context, surveillance of AC provides essential evidence to inform and evaluate antimicrobial stewardship (AMS) interventions.^4,7^ After a temporary decline during the first years of the COVID-19 pandemic, AC in the EU has returned above pre-pandemic levels.^8^ A similar pattern was observed in Italy, where AC decreased in 2020–2021 and subsequently increased in the following years.^9^ However, substantial heterogeneity persists across and within countries, reflecting differences in healthcare organization, prescribing practices, and patient case mix,^8,9^ underscoring the importance of region-specific analyses to guide tailored AMS strategies.

Lombardy, the most populous Italian region, represents a particularly relevant setting for AC surveillance. The region was the most heavily affected by the COVID-19 pandemic, especially during its initial phases,^10–12^ which profoundly disrupted healthcare delivery and prescribing behaviours.^13,14^ Hospitals represent a major source of AC, particularly of broad-spectrum and last-resort agents associated with the emergence and spread of AMR.^15^ Notably, AC varies substantially across hospital wards, with medical, surgical, and intensive care units showing distinct usage patterns reflecting their patient populations and clinical practices.^16–18^ Understanding inter-ward variations is crucial for identifying specific areas where AMS interventions can be most effectively targeted.

Given these premises, this study aimed to investigate temporal trends in in-hospital AC in Lombardy, Italy, between 2017 and 2024, stratified by ward type and by antibiotic group as defined in the WHO AWaRe classification, with particular focus on the impact of the COVID-19 pandemic.

## Methods

### Data sources

Data were retrieved from the administrative health databases of the Lombardy Region, Italy, routinely used for reimbursement purposes by public and private accredited hospitals. Specifically, two databases were analysed, collecting (i) purchases of antibiotics for use in inpatient hospital wards (hospital drug purchase records) and (ii) hospital bed days (hospital discharge forms).

Monthly purchase data of antibiotics for systemic use (ATC group J01) were obtained in kilograms per active ingredient and converted to DDDs utilizing the WHO Anatomical Therapeutic Chemical Classification System (ATC/DDD). Total sales were used as a proxy for AC as reported in previous studies^19,20^ and surveillance reports.^8^ Antibiotics were classified as Access, Watch, or Reserve according to the 2023 WHO AWaRe framework.^21^

Hospital wards were classified in medical units (MUs), surgical units (SUs), and intensive care units (ICUs).

The observation period spanned from 1 January 2017 to 31 December 2024 and was divided into four subperiods: pre–COVID-19 (January 2017–February 2020), COVID-19 onset (February–March 2020), COVID-19 (March 2020–May 2023), and post–COVID-19 (May 2023–December 2024).

### Outcomes

Four metrics of in-hospital AC were determined for each month and year of the observation period: (i) absolute AC, computed as the DDD per 100 bed days (for each WHO AWaRe class); (ii) relative AC, measured as the percentage of Access, Watch, and Reserve AC over the total [(DDD_class_/DDD_total_) × 100]; (iii) Access-to-Watch index, calculated as DDD_Access_/DDD_Watch_; (iv) Access-to-(Watch + Reserve) index, calculated as DDD_Access_/(DDD_Watch_ + DDD_Reserve_). These metrics were calculated overall and for each hospital ward type.

### Statistical analysis

Trends in each of the four AC metrics were described as relative differences (percent change from baseline) between 2017 and 2024 as well as in the four study subperiods.

The impact of the COVID-19 pandemic on absolute and relative AC was assessed using generalized linear models (GLMs). Expected monthly values were estimated from the pre–COVID-19 period (January 2017–February 2020) by fitting a model including month as a categorical variable to account for seasonality and year as a continuous variable to model the underlying temporal trend. The fitted model was then used to estimate the expected monthly values and the corresponding 95% confidence intervals from March 2020 to December 2024 under the assumption that pre-pandemic trends would have continued in the absence of the pandemic. Observed values during this period were compared with the corresponding expected values and expressed as percentage variation. Model adequacy was assessed through residual diagnostics, including the Shapiro-Wilk test for normality of residuals, Q-Q plots, and residual-*versus*-predicted plots.

All the statistical analyses were performed with SAS Studio version 9.04.

## Results

### Trends in absolute antibiotic consumption

**Figure 1.**
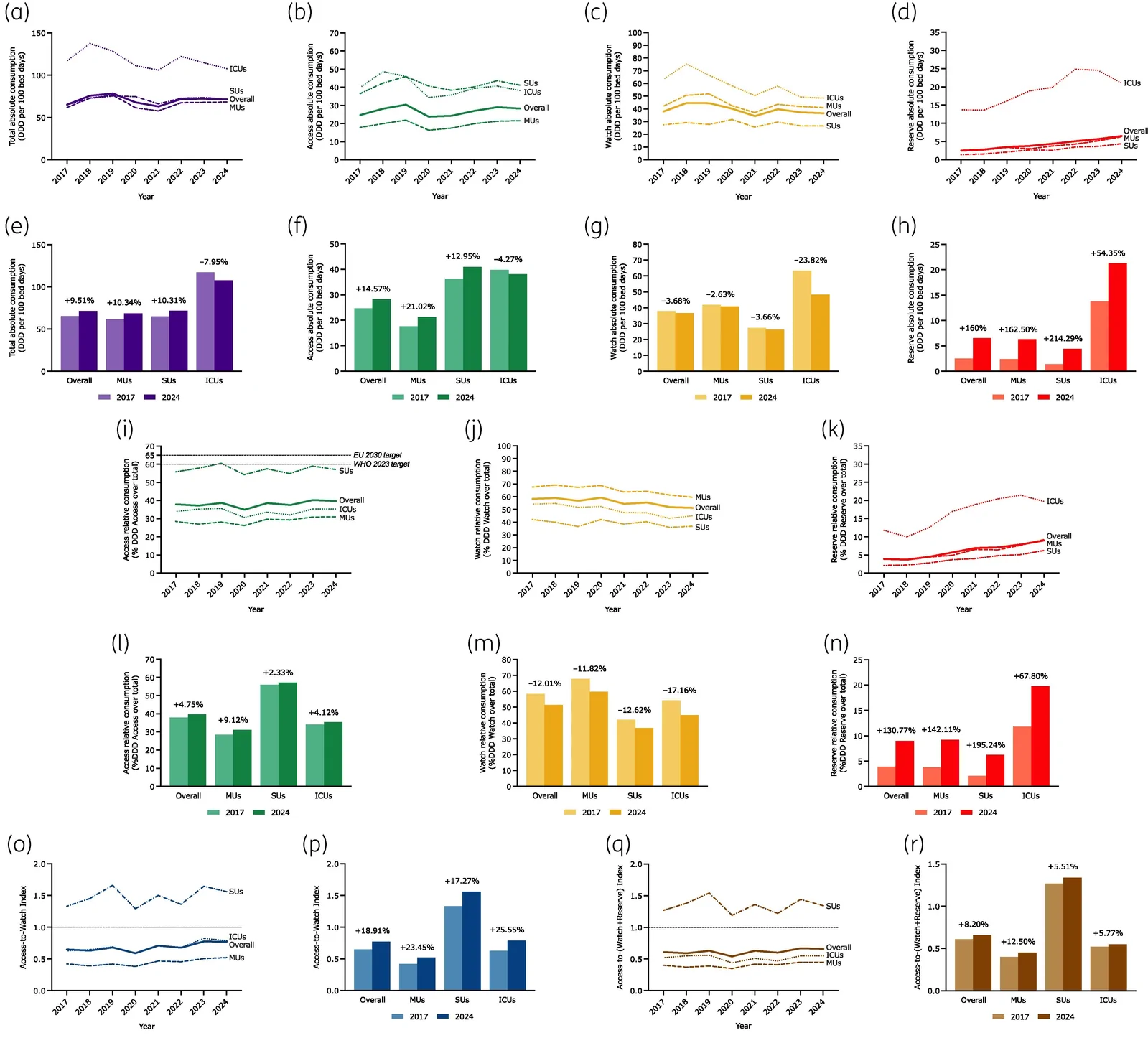
Yearly trends and percent changes between 2017 and 2024 in absolute antibiotic consumption (a–h), relative antibiotic consumption (i–n), Access-to-Watch index (o–p), and Access-to-(Watch + Reserve) index (q–r) overall, in medical units (MUs), surgical units (SUs), and intensive care units (ICUs). See Table S1 (absolute antibiotic consumption), Table S2 (relative antibiotic consumption), and Table S3 (Access-to-Watch index and Access-to-(Watch + Reserve) index) for detailed numerical data.

The analysis included data from 101 public and private accredited hospitals across Lombardy. During the study period, ICUs showed the highest total AC, with the greatest absolute consumption of Watch and Reserve antibiotics, whereas Access AC was higher in ICUs and SUs than in MUs (Figure 1a–d). Between 2017 and 2024, total AC increased in all wards except ICUs, where it declined. Absolute Access AC moderately increased across wards except in ICUs, while Watch AC decreased, particularly in ICUs. Conversely, Reserve AC markedly increased in all wards, with the largest rise observed in SUs (Figure 1e–h, Table S1 (available as Supplementary data at *JAC* Online)). A transient decline in total AC was observed in 2020–2021, mainly driven by reduced Access and Watch AC, despite a continued increase in Reserve AC (Figure 1a–d, Table S1).

Monthly analyses revealed heterogeneous temporal patterns across hospital wards and time periods (Table 1, Figure S1). During the pre–COVID-19 period, total AC increased overall and across all wards. Absolute Access AC increased overall, driven by increases in MUs and SUs despite a decline in ICUs; Watch AC increased in MUs and ICUs but decreased in SUs, resulting in an overall rise; Reserve AC increased markedly across all wards. At the COVID-19 onset, total AC decreased overall, with reductions in MUs and ICUs despite an increase in SUs. Similarly, Access, Watch, and Reserve AC declined overall: Access decreased in MUs and SUs but slightly increased in ICUs; Watch decreased in MUs and ICUs but increased in SUs; and Reserve decreased in MUs and ICUs while rising in SUs. During the COVID-19 period, total AC increased overall, driven by rises in MUs and SUs despite a decline in ICUs. Absolute Access AC increased, whereas Watch AC decreased across wards. Reserve AC increased overall, with marked rises in MUs and ICUs despite a reduction in SUs. In the post–COVID-19 period, total AC increased overall, driven by a rose in MUs despite slight declines in SUs and ICUs. Absolute Access AC decreased overall and in most wards, except for a modest increase in MUs, whereas both Watch and Reserve AC increased across all wards.

**Table 1.** Monthly trends in antibiotic consumption metrics. See Figures S1–S3 for a detailed graphical representation

AWaRe antibiotic class	Ward type	DDD per 100 bed days					Relative difference (%)			
		Jan 17	Feb 20	Mar 20	May 23	Dec 24	Jan 17 → Feb 20	Feb 20 → Mar 20	Mar 20 → May 23	May 23 → Dec 24
*Absolute antibiotic consumption*										
Total	Overall	69.1	79.4	66.5	71.5	77.3	+14.92%	−16.25%	+7.52%	+8.11%
	MUs	67.4	78.2	54.6	65.5	75.5	+16.03%	−30.18%	+19.96%	+15.27%
	SUs	65.5	74.7	94.4	75.7	73.9	+14.05%	+26.37%	−19.83%	−2.38%
	ICUs	119	129	108	121.6	118.3	+8.4%	−16.28%	+12.59%	−2.71%
Access	Overall	22.8	28.46	17.94	31.83	29.69	+24.83%	−36.94%	+77.46%	−6.72%
	MUs	16.7	20.66	9.88	22.39	23.09	+23.71%	−52.17%	+126.6%	+3.13%
	SUs	33.33	44.36	41.76	47.52	41.39	+33.09%	−5.86%	+13.8%	−12.90%
	ICUs	37.23	29.63	31.54	53.51	41.09	−20.41%	+6.45%	+69.7%	−23.21%
Watch	Overall	44.37	47.21	45.12	34.7	40.29	+6.4%	−4.43%	−23.1%	+16.11%
	MUs	48.81	54.35	42.29	38.51	45.24	+11.35%	−22.20%	−8.9%	+17.47%
	SUs	30.87	27.81	48.36	24.9	27.5	−9.92%	+73.89%	−48.5%	+10.44%
	ICUs	75.01	80.05	64.79	47.43	55.06	+6.72%	−19.07%	−26.8%	+16.08%
Reserve	Overall	1.95	3.69	3.47	4.99	7.29	+89.23%	−5.96%	+43.9%	+46.09%
	MUs	1.88	3.19	2.46	4.59	7.14	+69.68%	−22.91%	+86.6%	+55.56%
	SUs	1.42	2.5	4.26	3.28	5.04	+76.06%	+70.40%	−23%	+53.66%
	ICUs	6.78	19.31	11.68	20.67	22.18	+184.7%	−39.52%	+76.95%	+7.3%

AWaRe antibiotic class	Ward type	% DDD_total_					Relative difference (%)			
		Jan 17	Feb 20	Mar 20	May 23	Dec 24	Jan 17 → Feb 20	Feb 20 → Mar 20	Mar 20 → May 23	May 23 → Dec 24
*Relative antibiotic consumption*										
Access	Overall	33	35.9	27	44.5	38.4	+8.79%	−24.79%	+64.8%	−13.71%
	MUs	24.8	26.4	18.1	34.2	30.6	+6.45%	−31.44%	+88.95%	−10.53%
	SUs	50.8	59.4	44.2	62.8	56	+16.93%	−25.59%	+42.2%	−10.83%
	ICUs	31.3	23	29.2	44	34.7	−26.52%	+26.96%	+50.7%	−21.14%
Watch	Overall	64.2	59.5	67.8	48.5	52.1	−7.32%	+13.95%	−28.5%	+7.42%
	MUs	72.4	69.5	77.4	58.8	59.9	−4.01%	+11.37%	−24%	+1.87%
	SUs	47	37.2	51.2	32.9	37.2	−20.85%	+37.63%	−35.7%	+13.07%
	ICUs	63	62.1	60	39	46.5	−1.43%	−3.38%	−35%	+19.23%
Reserve	Overall	2.8	4.7	5.2	7	9.4	+67.86%	+10.64%	+34.6%	+34.29%
	MUs	2.8	4.1	4.5	7	9.5	+46.43%	+9.76%	+55.6%	+35.71%
	SUs	2.2	3.3	4.5	4.3	6.8	+50%	+36.36%	−4.44%	+58.14%
	ICUs	5.7	15	10.8	17	18.7	+163.2%	−28%	+57.4%	+10%

Index	Ward type	Index value					Relative difference (%)			
		Jan 17	Feb 20	Mar 20	May 23	Dec 24	Jan 17 → Feb 20	Feb 20 → Mar 20	Mar 20 → May 23	May 23 → Dec 24
*Access-to-Watch index and Access-to-(Watch* *+* *Reserve) index*										
A/W-I	Overall	0.51	0.60	0.40	0.92	0.74	+17.65%	−33.33%	+130%	−19.57%
	MUs	0.34	0.38	0.23	0.58	0.51	+11.76%	−39.47%	+152.2%	−12.07%
	SUs	1.08	1.60	0.86	1.91	1.51	+48.15%	−46.25%	+122.1%	−20.94%
	ICUs	0.50	0.37	0.49	1.13	0.75	−26%	+32.43%	+130.6%	−33.63%
A/(W + R)-I	Overall	0.49	0.56	0.37	0.80	0.62	+14.29%	−33.93%	+116.22%	−22.50%
	MUs	0.33	0.36	0.22	0.52	0.44	+33.33%	−38.89%	+136.36%	−15.38%
	SUs	1.03	1.46	0.79	1.69	1.27	+41.75%	−45.89%	+113.92%	−24.85%
	ICUs	0.46	0.30	0.41	0.79	0.53	−34.78%	+36.67%	+92.68%	−32.91%

*DDD*, defined daily doses; *MUs*, medical units; *SUs*, surgical units; *ICUs*, intensive care units; *A/W-I*, Access-to-Watch index; *A/(W* *+* *R)-I*, Access-to-(Watch + Reserve) index

### Trends in relative antibiotic consumption

Between 2017 and 2024, relative Access AC was highest in SUs and lowest in MUs, whereas Watch AC showed the opposite pattern; relative Reserve AC was highest in ICUs, followed by MUs and SUs (Figure 1i–k). The WHO 2023 target of ≥60% Access antibiotics was not achieved overall, although SUs approached this threshold and exceeded it in 2019; the EU 2030 target of ≥65% Access antibiotics was not reached in any ward (Figure 1i). Over the study period, relative Access AC moderately increased, while Watch AC declined across all wards. Conversely, relative Reserve AC markedly increased, particularly in SUs (Figure 1l–n, Table S2).

Distinct monthly trends were observed across periods and wards (Table 1, Figure S2). During the pre–COVID-19 period, relative Access AC increased overall, driven by rises in MUs and SUs despite a decline in ICUs. Conversely, relative Watch AC decreased overall and across all wards, whereas Reserve AC increased in all hospital wards. At the COVID-19 onset, relative Access AC decreased overall, with reductions in MUs and SUs despite an increase in ICUs. Conversely, relative Watch AC increased overall, driven by rises in MUs and SUs despite a slight decline in ICUs. Relative Reserve AC also rose overall, with increases in MUs and SUs and a reduction in ICUs. During the COVID-19 period, relative Access AC increased overall and across all wards, whereas Watch AC decreased. Relative Reserve AC increased overall, driven by rises in MUs and ICUs despite a slight decline in SUs. In the post–COVID-19 period, Access AC decreased across all wards, while both Watch and Reserve AC increased consistently.

### Trends in Access-to-Watch index and Access-to-(Watch + Reserve) index

During the study period, the Access-to-Watch index was lowest in MUs and highest in SUs, where it consistently remained above 1 (Figure 1o). Between 2017 and 2024, the index moderately increased across all wards (Figure 1p). Monthly trends varied across periods: it increased overall during the pre–COVID-19 period despite a decline in ICUs, decreased at COVID-19 onset due to reductions in MUs and SUs despite an increase in ICUs, rose during the COVID-19 period, and declined across all wards post-pandemic (Table 1, Figure S3).

Trends in the Access-to-(Watch + Reserve) index were consistent with those of the Access-to-Watch index (Figure 1q–r, Table 1, Figure S3).

### Impact of the COVID-19 pandemic on absolute and relative antibiotic consumption

Compared with the expected counterfactual scenario in the absence of COVID-19, relevant changes in in-hospital AC patterns emerged from March 2020 onwards.

**Figure 2.**
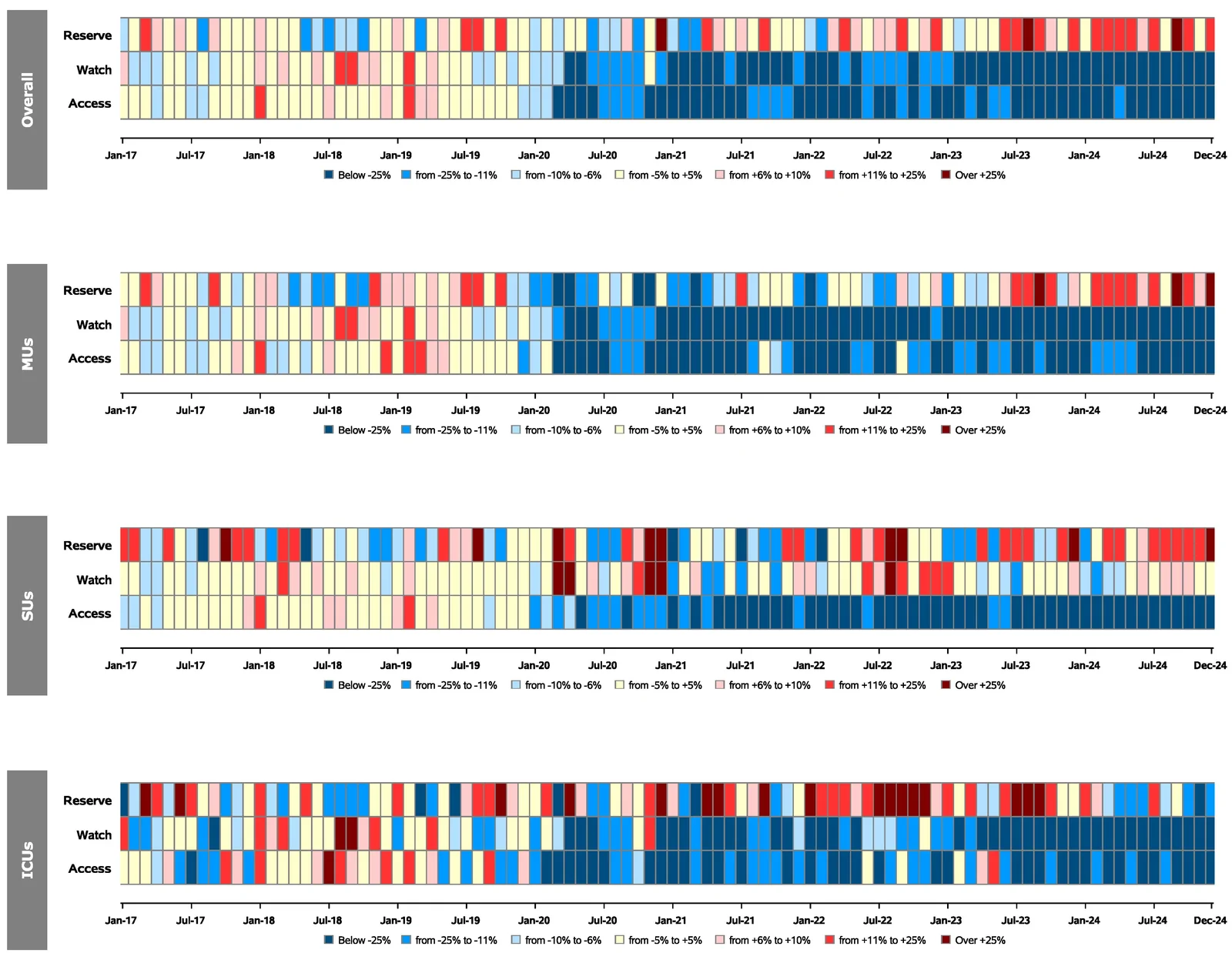
Percent changes in observed monthly absolute antibiotic consumption as compared to the expected values in the absence of COVID-19 as predicted by generalized linear models (GLMs). Cool colours (different shades of blue) indicate a reduction (lower-than-expected observed levels), while warm colours (yellow, pink, red) an increase (higher-than-expected observed levels). See Figure S4 for detailed monthly trends in observed *versus* expected absolute antibiotic consumption according to WHO AWaRe class and ward type. See Table S4 for the normality test of residuals and R^2^ of the GLMs, as well as Figure S6 and Figure S7 for Q-Q plots and residual-*versus*-predicted plots, respectively.

For absolute AC, Access and Watch consumption remained generally below expected levels throughout the observation period, whereas Reserve AC exceeded expected values, particularly from June 2023. In MUs, Access and Watch AC were lower than expected, while Reserve AC shifted from lower-than-expected levels during the early pandemic (until June 2021) to higher-than-expected levels from June 2023. In SUs, Access AC was lower, whereas Watch and Reserve AC were higher than expected. In ICUs, Access and Watch AC were lower, while Reserve AC was higher than expected (Figure 2, Figure S4).

**Figure 3.**
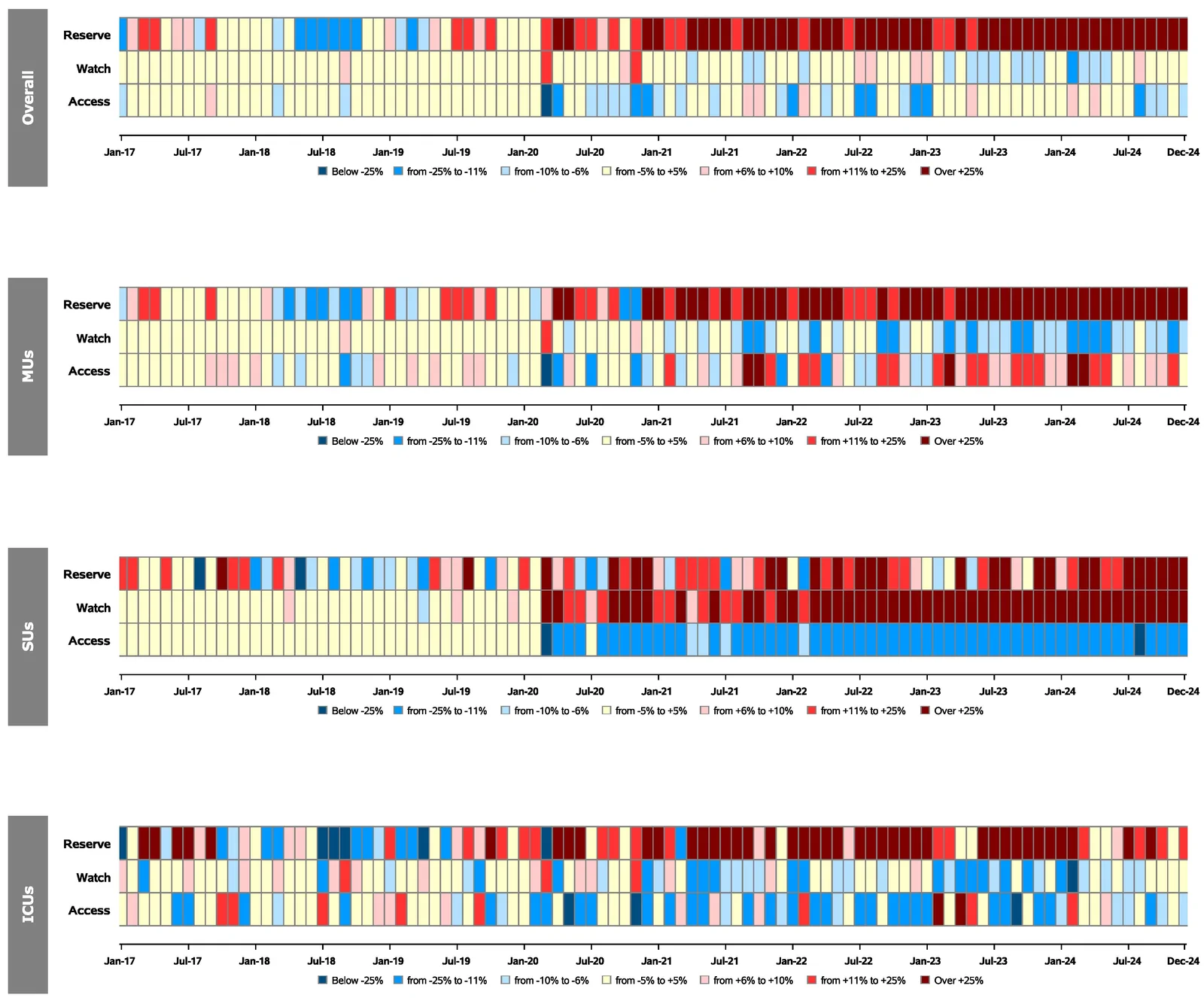
Percent changes in observed monthly relative antibiotic consumption as compared to the expected values in the absence of COVID-19 as predicted by generalized linear models (GLMs). Cool colours (different shades of blue) indicate a reduction (lower-than-expected observed levels), while warm colours (yellow, pink, red) an increase (higher-than-expected observed levels). See Figure S5 for detailed monthly trends in observed *versus* expected relative antibiotic consumption according to WHO AWaRe class and ward type. See Table S4 for the normality test of residuals and R^2^ of the GLMs, as well as Figures S6 and S7 for Q-Q plots and residual-*versus*-predicted plots, respectively.

For relative AC, Access and Watch consumption remained broadly consistent with expected trends overall, whereas Reserve AC was consistently higher than expected. Ward-specific analyses showed lower Access and higher Watch and Reserve AC in SUs, higher Reserve AC throughout in ICUs, and in MUs a late increase in Access with a decline in Watch from September 2023, alongside persistently higher-than-expected Reserve AC (Figure 3, Figure S5).

## Discussion

In this retrospective analysis of regional administrative data, we described temporal trends in in-hospital consumption of antibiotics for systemic use in Lombardy, Italy, between 2017 and 2024, stratified by ward type and by WHO AWaRe group, with a particular focus on the impact of the COVID-19 pandemic.

In absolute terms, ICUs consistently showed the highest AC across all AWaRe classes, reflecting the clinical complexity of cases and the higher risk of healthcare-associated infections in this setting.^22^ However, when assessing relative AC, distinct patterns emerged across ward types. Access antibiotics accounted for the largest proportion of total AC in SUs, consistent with their prominent role in preoperative prophylaxis, which represents a relevant source of AC in these wards.^23,24^ Conversely, Watch antibiotics predominated in MUs, likely reflecting the broader range of empiric treatments required for community-acquired and healthcare-associated infections in patients with diverse comorbidities. Reserve antibiotics represented the highest relative share in ICUs, in line with the frequent need for coverage against MDR pathogens in critically ill patients.^25^

Between 2017 and 2024, total AC increased in Lombardy hospitals. This trend is consistent with recent data from the Italian Medicines Agency, which reported an 8.2% rise in in-hospital AC across Italy between 2019 and 2024, albeit with some geographical variability. The largest increases were observed in Northern (+10%) and Central (+7.2%) regions, whereas Southern Italy showed a smaller increase (+5.5%).^9^

The rise in total AC in Lombardy was accompanied by a shift in the distribution across AWaRe categories, characterized by an increase in Access and a decline in Watch AC. These shifts resulted in an increase of both Access-to-Watch and Access-to-(Watch + Reserve) indexes, reflecting an overall improving trend in in-hospital AC patterns. According to the European Surveillance of Antimicrobial Consumption Network (ESAC-Net), Access antibiotics accounted for 60.3% of total antibiotic consumption across EU countries in 2024, remaining below the 2030 target of >65%, with an even lower proportion in hospitals (45.3%).^8^ In Italy, national data from the Italian Medicines Agency showed a lower consumption of Access antibiotics in 2024, accounting for 54.8% of reimbursed prescriptions overall and 36.2% in hospitals.^9^ Importantly, in keeping with these national data, despite an overall increase in Access AC in 2017–2024, neither the EU 2030 65% target nor the WHO 2023 60% target was achieved in Lombardy hospitals, with the highest proportion (40.3%) recorded in 2023. Similarly, although both Access-to-Watch and Access-to-(Watch + Reserve) indexes increased over time, they constantly remained below 1 overall, indicating persistent overuse of Watch agents. Interestingly, marked ward-level differences were observed, with the lowest relative Access AC and indexes in MUs, whereas SUs approached or exceeded the WHO 2023 60% target and consistently maintained both indexes above 1. This pattern may indicate a more appropriate antibiotic use in SUs and could be partially explained by the routine use of Access antibiotics for preoperative surgical prophylaxis.^23,24^ Overall, these findings highlight a persistent gap between AC patterns in Lombardy hospitals and international AMS targets, particularly in MUs and ICUs. However, these data may also reflect the higher burden of MDR pathogens in Italy—including third-generation cephalosporin-resistant *Escherichia coli*, methicillin-resistant *Staphylococcus aureus* (MRSA), and carbapenem-resistant *Klebsiella pneumoniae*—compared with the European average,^3^ which limits the feasibility of prioritizing Access antibiotics.

Of note, in-hospital Reserve AC rose markedly in 2017–2024, particularly in SUs, where absolute AC grew by 162.5% and relative AC by 195.2%. Similar trends have been reported at both the European and national levels. According to ESAC-Net, Reserve AC continued to increase across the EU in 2024.^8^ Likewise, the Italian Medicines Agency documented a sustained increase in in-hospital Reserve AC from 2016 onwards. Although this upward trend was consistent across all geographical areas until 2022, Reserve AC subsequently declined in Northern and Central Italy, largely driven by reduced daptomycin consumption, while continuing to increase in Southern Italy in 2024.^9^ This overall rise may reflect a growing need to appropriately treat MDR infections in recent years, as supported by European data showing a marked increase between 2019 and 2024 in the estimated incidence of BSIs caused by carbapenem-resistant *K. pneumoniae*, third-generation cephalosporin-resistant and carbapenem-resistant *E. coli*, and vancomycin-resistant *Enterococcus faecium* (VRE).^3^ However, regional surveillance data from Lombardy suggest a more heterogeneous picture: while the proportion of VRE increased between 2017 and 2024, MRSA, carbapenem-resistant *K. pneumoniae* and *P. aeruginosa* decreased, and carbapenem-resistant *E. coli* and *Acinetobacter* spp. remained substantially stable.^26^ Therefore, the marked increase in Reserve AC observed in Lombardy cannot be explained solely by a parallel increase in resistance patterns and may also reflect changes in prescribing practices, including broader empirical use of these agents, together with evolving therapeutic options. These findings underscore the need for targeted AMS interventions aimed at ensuring appropriate indications and optimizing the duration of Reserve antibiotic therapy.

Interestingly, distinct monthly trends in AC metrics were observed across study periods, likely reflecting the interplay between AMS efforts and external pressures such as COVID-19. Before the pandemic, despite a growth in total AC, increasing Access-to-Watch and Access-to-(Watch + Reserve) indexes—driven by higher Access and lower Watch consumption—suggested progressive alignment with AMS goals. However, this favourable trajectory was reversed at COVID-19 onset, when, despite an overall reduction of total AC in line with other reports,^8,9,20,27–29^ a reduction in both indexes was registered, likely reflecting disruption of AMS practices.^30,31^ This disruption was likely driven by diagnostic uncertainty and frequent empirical treatment of suspected bacterial coinfections or superinfections with broad-spectrum antibiotics.^14,30,31^ During the subsequent pandemic period, total AC rose again, with a recovery of Access and a decline in Watch AC; this shift led to improved indexes, possibly reflecting the gradual adaptation of clinical practice to accumulating scientific evidence and the reimplementation of AMS programmes. However, the post-pandemic decline in both indexes accompanied by a continued growth in total AC raises concerns regarding the long-term sustainability of AMS achievements. Notably, while Access and Watch consumption fluctuated over time, Reserve AC grew consistently across periods and wards. This trend may reflect multiple factors, including the growing burden of MDR pathogens,^3^ the wider availability of new Reserve agents in the last years,^32^ and potential gaps in AMS efforts, which may have prioritized monitoring of the most commonly used Watch antibiotics over Reserve agents, which are typically prescribed as last-resort treatments on a case-by-case basis without being strictly subjected to systematic auditing.

The impact of COVID-19 on in-hospital AC trajectories was confirmed by comparing observed trends from March 2020 onwards with the expected counterfactual scenario based on pre-pandemic data. Overall, while Access and Watch AC remained below or similar to expected levels, Reserve AC consistently exceeded expectations across wards, indicating a greater-than-anticipated increase in consumption of these agents. Interestingly, in SUs, relative Access AC was lower and relative Watch AC higher than expected, suggesting that, had COVID-19 not occurred, the increase in Access and the decline in Watch AC would have been more pronounced. These long-lasting disruptions observed during and following the pandemic may reflect multiple interconnected factors, including enduring structural changes in hospital organization, delayed reactivation of AMS programmes, and behavioural shifts in prescribing practices.^33^

This study has some limitations. Firstly, the reliance on administrative databases without patient-level data limited the assessment of differences in in-hospital AC according to demographic features (i.e. age and sex) and clinical indications (e.g. medical/surgical prophylaxis *versus* therapeutic use, or treatment of community-acquired *versus* healthcare-associated infections). Additionally, as in previous studies^19,20^ and official EU surveillance reports,^8^ AC was estimated using sales data, a standard approach for population-level surveillance; however, these data may not accurately reflect actual antibiotic use at the patient level, as purchased quantities may differ from administered doses. Moreover, DDD-based estimates may not accurately capture antibiotic exposure in ICUs, where dosing is frequently individualized according to disease severity and/or organ dysfunction, potentially affecting comparisons across ICUs with different case mixes due to over- or underestimation; nevertheless, DDD remains a consistent and reproducible metric that enables comparisons across hospital wards and over time. Lastly, while understanding in-hospital AC is critical given its role as a major driver of healthcare-associated infections caused by MDR pathogens and its importance in guiding targeted AMS interventions in settings with high Watch and Reserve AC, the community remains the predominant source of total AC.^8^ Therefore, future research should extend the analysis to community settings to provide a more comprehensive understanding of overall AC in Lombardy and to support the development of AMS strategies beyond the hospital setting.

### Conclusions

In conclusion, this study showed that, despite a surge in total AC in Lombardy hospitals between 2017 and 2024, relative Access AC rose while that of Watch antibiotics declined, resulting in an overall increase of both Access-to-Watch and Access-to-(Watch + Reserve) indexes, which indicates a gradual progress towards more appropriate antibiotic use. Of note, these favourable trends were temporarily disrupted during the COVID-19 pandemic, emphasizing the vulnerability of AMS programmes to systemic disruptions and underscoring the importance of embedding them as a structural component of hospital governance and pandemic preparedness planning. Nevertheless, despite these improvements, the WHO 2023 target of at least 60% Access antibiotics was neither achieved nor closely approached, and Access-to-Watch and Access-to(Watch + Reserve) indexes constantly remained below 1, except for SUs. Furthermore, a downward trend in these indexes was registered following the pandemic, raising concerns about long-term AMS sustainability. Moreover, in-hospital Reserve AC steadily increased across periods and wards, exceeding the expected levels in the absence of COVID-19, potentially reflecting multiple concurrent factors, including the increasing complexity of treating MDR infections, evolving therapeutic options, and possible gaps in AMS oversight for such last-resort agents, which may have been exacerbated by the pandemic.

These findings highlight the need to further strengthen existing AMS programmes within the framework of the Italian National Action Plan on Antimicrobial Resistance (PNCAR) and its regional implementation.^34^ Although multidisciplinary AMS teams and regional surveillance systems have progressively been established across Italy, their organization, resources, and implementation remain heterogeneous. Our findings suggest that particular emphasis should be placed on routine ward-level feedback based on AWaRe indicators, closer monitoring of Reserve antibiotics prescribing, and integration of AC data with microbiological surveillance and patient-level clinical information to support assessment of prescribing appropriateness. Aligning AMS targets with European and WHO benchmarks, together with sustained investment in workforce training, diagnostic capacity, and surveillance systems, may further strengthen the effectiveness and resilience of AMS programmes in the face of future systemic shocks within the Italian healthcare system and beyond.

## Supplementary Material

dkag314_Supplementary_Data

## Data Availability

Data that support the findings of this study are available by the Lombardy Region upon reasonable request, but restrictions apply to the availability of these data and codes, which were used under license for the current study, and so they are not publicly available.

## Supplementary data

Figures S1 to S7 and Tables S1 to S4 are available as Supplementary data at *JAC* Online.
