# Supplementary material for: In-hospital antibiotic consumption in Lombardy, Italy (2017–2024): a retrospective analysis of regional administrative data
Source: J Antimicrob Chemother. 2026 Sep 7;81(10):dkag314. doi: 10.1093/jac/dkag314 (PMC13546790; doi:10.1093/jac/dkag314)
Supplement: Supplementary file 1 [file dkag314_supplementary_data.docx]

**Supplementary material**

**Table S1.** Yearly trends in absolute antibiotic consumption (DDD per 100 bed days).

| **AWaRe antibiotic class** | **Ward type** | **2017** | **2018** | **2019** | **2020** | **2021** | **2022** | **2023** | **2024** |
| --- | --- | --- | --- | --- | --- | --- | --- | --- | --- |
| Total | Overall | 65.2 | 75.6 | 78.4 | 67.8 | 63 | 71.8 | 72 | 71.4 |
|  | MUs | 61.9 | 72.9 | 76.5 | 61.4 | 57.8 | 67.5 | 67.9 | 68.3 |
|  | SUs | 65 | 72.7 | 75.4 | 74.7 | 66.1 | 72.8 | 73.5 | 71.7 |
|  | ICUs | 117 | 137.7 | 128.5 | 111.3 | 106.1 | 122.2 | 114.7 | 107.7 |
| Access | Overall | 24.7 | 28.2 | 30.4 | 23.8 | 24.3 | 27 | 29 | 28.3 |
|  | MUs | 17.6 | 19.7 | 21.6 | 16.1 | 17.2 | 19.7 | 21 | 21.3 |
|  | SUs | 36.3 | 42.1 | 45.7 | 40.5 | 38.1 | 40 | 43.4 | 41 |
|  | ICUs | 39.8 | 48.6 | 45.9 | 34.2 | 35.6 | 39.3 | 40.6 | 38.1 |
| Watch | Overall | 38 | 44.6 | 44.5 | 40.2 | 34.3 | 39.8 | 37.3 | 36.6 |
|  | MUs | 41.9 | 50.5 | 51.6 | 42.3 | 36.9 | 43.4 | 41.7 | 40.8 |
|  | SUs | 27.3 | 29 | 27.5 | 31.4 | 25.4 | 29.4 | 26.4 | 26.3 |
|  | ICUs | 63.4 | 75.3 | 66.5 | 58.2 | 50.5 | 57.9 | 49.4 | 48.3 |
| Reserve | Overall | 2.5 | 2.8 | 3.5 | 3.8 | 4.4 | 5.1 | 5.7 | 6.5 |
|  | MUs | 2.4 | 2.7 | 3.4 | 3 | 3.8 | 4.3 | 5.2 | 6.3 |
|  | SUs | 1.4 | 1.6 | 2.1 | 2.7 | 2.6 | 3.5 | 3.7 | 4.4 |
|  | ICUs | 13.8 | 13.7 | 16.2 | 19 | 20 | 25 | 24.7 | 21.3 |
| **Legend:** *MUs,* medical units; *SUs,* surgical units; *ICUs,* intensive care units | | | | | | | | | |

**Table S2.** Yearly trends in relative antibiotic consumption (% DDD_total_).

| **AWaRe antibiotic class** | **Ward type** | **2017** | **2018** | **2019** | **2020** | **2021** | **2022** | **2023** | **2024** |
| --- | --- | --- | --- | --- | --- | --- | --- | --- | --- |
| Access | Overall | 37.9 | 37.2 | 38.7 | 35 | 38.6 | 37.5 | 40.3 | 39.7 |
|  | MUs | 28.5 | 27 | 28.2 | 26.2 | 29.7 | 29.3 | 30.9 | 31.1 |
|  | SUs | 55.9 | 57.9 | 60.6 | 54.3 | 57.6 | 54.9 | 59.1 | 57.2 |
|  | ICUs | 34 | 35.3 | 35.7 | 30.7 | 33.6 | 32.1 | 35.4 | 35.4 |
| Watch | Overall | 58.3 | 59 | 56.8 | 59.3 | 54.4 | 55.4 | 51.8 | 51.3 |
|  | MUs | 67.7 | 69.3 | 67.4 | 68.9 | 63.8 | 64.3 | 61.4 | 59.7 |
|  | SUs | 42 | 39.9 | 36.5 | 42 | 38.4 | 40.3 | 35.9 | 36.7 |
|  | ICUs | 54.2 | 54.7 | 51.7 | 52.3 | 47.5 | 47.4 | 43 | 44.9 |
| Reserve | Overall | 3.9 | 3.7 | 4.5 | 5.7 | 6.9 | 7.1 | 7.9 | 9 |
|  | MUs | 3.8 | 3.7 | 4.4 | 4.9 | 6.5 | 6.4 | 7.7 | 9.2 |
|  | SUs | 2.1 | 2.2 | 2.8 | 3.7 | 4 | 4.8 | 5.1 | 6.2 |
|  | ICUs | 11.8 | 10 | 12.6 | 17 | 18.9 | 20.5 | 21.5 | 19.8 |
| **Legend:** *MUs,* medical units; *SUs,* surgical units; *ICUs,* intensive care units | | | | | | | | | |

**Table S3.** Yearly trends in Access-to-Watch index (A/W-I) and Access-to-(Watch+Reserve) index [A/(W+R)-I].

| **Index** | **Ward type** | **2017** | **2018** | **2019** | **2020** | **2021** | **2022** | **2023** | **2024** |
| --- | --- | --- | --- | --- | --- | --- | --- | --- | --- |
| A/W-I | Overall | 0.65 | 0.63 | 0.68 | 0.59 | 0.71 | 0.68 | 0.78 | 0.77 |
|  | MUs | 0.42 | 0.39 | 0.42 | 0.38 | 0.47 | 0,45 | 0.50 | 0.52 |
|  | SUs | 1.33 | 1.45 | 1.66 | 1.29 | 1.50 | 1.36 | 1.65 | 1.56 |
|  | ICUs | 0.63 | 0.65 | 0.69 | 0.59 | 0.71 | 0.68 | 0.82 | 0.79 |
| A/(W+R)-I | Overall | 0.61 | 0.59 | 0.63 | 0.54 | 0.63 | 0.60 | 0.67 | 0.66 |
|  | MUs | 0.40 | 0.37 | 0.39 | 0.35 | 0.42 | 0.41 | 0.45 | 0.45 |
|  | SUs | 1.27 | 1.38 | 1.54 | 1.19 | 1.36 | 1.22 | 1.44 | 1.34 |
|  | ICUs | 0.52 | 0.55 | 0.56 | 0.44 | 0.51 | 0.47 | 0.55 | 0.55 |
| **Legend:** *MUs,* medical units; *SUs,* surgical units; *ICUs,* intensive care units | | | | | | | | | |

**Table S4.** Test for normality of residuals and R^2^ of the generalised linear models (GLMs) used for the analysis of the impact of the COVID-19 pandemic on absolute and relative antibiotic consumption. The Shapiro-Wilk test do not indicate significant departures from normality, supporting the adequacy of the model fit.

| **Outcome** | **Ward type** | **Test for normality of residuals* (*P* value)** | **R^2^** |
| --- | --- | --- | --- |
| DDD Access per 100 bed days | Overall | 0.1840 | 0.75 |
|  | MUs | 0.1736 | 0.71 |
|  | SUs | 0.5750 | 0.75 |
|  | ICUs | 0.3701 | 0.39 |
| DDD Watch per 100 bed days | Overall | 0.3031 | 0.60 |
|  | MUs | 0.0972 | 0.65 |
|  | SUs | 0.8503 | 0.49 |
|  | ICUs | 0.0526 | 0.35 |
| DDD Reserve per 100 bed days | Overall | 0.6074 | 0.79 |
|  | MUs | 0.1249 | 0.77 |
|  | SUs | 0.2143 | 0.67 |
|  | ICUs | 0.3087 | 0.33 |
| % DDD Access | Overall | 0.2415 | 0.63 |
|  | MUs | 0.3837 | 0.55 |
|  | SUs | 0.4717 | 0.80 |
|  | ICUs | 0.7950 | 0.43 |
| % DDD Watch | Overall | 0.0880 | 0.61 |
|  | MUs | 0.3210 | 0.55 |
|  | SUs | 0.5261 | 0.85 |
|  | ICUs | 0.8130 | 0.48 |
| % DDD Reserve | Overall | 0.5646 | 0.48 |
|  | MUs | 0.2616 | 0.52 |
|  | SUs | 0.7400 | 0.55 |
|  | ICUs | 0.4731 | 0.20 |
| * Shapiro-Wilk's normality test | | | |

**Figure S1.** Monthly trends in relative in-hospital antibiotic consumption overall, in medical units (MUs), surgical units (SUs), and intensive care units (ICUs) for Access, Watch, and Reserve antibiotics. The blue lines mark the study subperiods (pre–COVID-19: January 2017–February 2020; COVID-19 onset: February–March 2020; COVID-19: March 2020–May 2023; post–COVID-19: May 2023–December 2024), the red line marks the WHO 2023 target of at least 60% Access antibiotics in total antibiotic consumption, while the purple line marks the EU 2030 target of at least 65% Access antibiotics in total antibiotic consumption.

**Figure S2.** Monthly trends in relative in-hospital antibiotic consumption overall, in medical units (MUs), surgical units (SUs), and intensive care units (ICUs) for Access, Watch, and Reserve antibiotics. The blue lines mark the study subperiods (pre–COVID-19: January 2017–February 2020; COVID-19 onset: February–March 2020; COVID-19: March 2020–May 2023; post–COVID-19: May 2023–December 2024), the red line marks the WHO 2023 target of at least 60% Access antibiotics in total antibiotic consumption, while the purple line marks the EU 2030 target of at least 65% Access antibiotics in total antibiotic consumption.

**Figure S3.** Monthly trends in Access-to-Watch index (**A**) and Access-to-(Watch+Reserve) index (**B**) overall, in medical units (MUs), surgical units (SUs), and intensive care units (ICUs). The blue lines mark the study subperiods (pre–COVID-19: January 2017–February 2020; COVID-19 onset: February–March 2020; COVID-19: March 2020–May 2023; post–COVID-19: May 2023–December 2024).

**Figure S4.** Monthly trends in observed *versus* expected absolute antibiotic consumption according to WHO AWaRe class and ward type.

**Figure S5.** Monthly trends in observed *versus* expected relative antibiotic consumption according to WHO AWaRe class and ward type.

**Figure S6.** Q-Q plots of residuals of the generalised linear models (GLMs) used for the analysis of the impact of the COVID-19 pandemic on absolute and relative antibiotic consumption. Q-Q plots show that the residuals are approximately aligned with the reference line, supporting the adequacy of the model fit.

**Figure S7.** Residual-*versus*-predicted plots of the generalised linear models (GLMs) used for the analysis of the impact of the COVID-19 pandemic on absolute and relative antibiotic consumption. Residual-*versus*-predicted plots do not reveal systematic patterns suggestive of non-linearity or heteroscedasticity, supporting the adequacy of the model fit.
